# Total hip arthroplasty after failed transtrochanteric rotational osteotomy for osteonecrosis of the femoral head: analysis of three-dimensional morphological features

**DOI:** 10.1186/s12891-024-07299-z

**Published:** 2024-03-04

**Authors:** Masamichi Onaga, Satoshi Nakasone, Masato Ishihara, Takahiro Igei, Fumiyuki Washizaki, Sakura Kuniyoshi, Kotaro Nishida

**Affiliations:** 1 Department of Orthopedic Surgery, Graduate School of Medicine, University of the Ryukyus, 207 Aza-Uehara, Nishihara Cho, Nakagami-Gun, Okinawa, 9030215 Japan; 2 Department of Orthopedic Surgery, Nakagami Hospital, 610 Noborikawa, Okinawa, Okinawa 9042195 Japan

**Keywords:** Osteonecrosis of the femoral head, Total hip arthroplasty, Transtrochanteric rotational osteotomy, Proximal femoral deformity, Stem malalignment, Three-dimensional analysis

## Abstract

**Background:**

In total hip arthroplasty (THA) after failed transtrochanteric rotational osteotomy (TRO) for osteonecrosis of the femoral head (ONFH), deformity of the proximal femur has been reported to affect stem placement. The aims of this study were to evaluate the morphological changes in the proximal femur, muscle atrophy, and soft tissue thickening in THA after TRO and the clinical outcomes.

**Methods:**

The TRO group included 17 patients (18 hips) who underwent THA after failed TRO. The control group included 21 patients (28 hips) who underwent primary THA for ONFH. To evaluate the deformity of the proximal femur before THA, we measured the anteroposterior and mediolateral diameters of the femur on computed tomographic slices 5 mm proximal to the lesser trochanter. To evaluate muscle atrophy and soft tissue thickening, we measured the thicknesses of the psoas major, iliac, and gluteus medius muscles and the anterior capsule of the hip joint.

**Results:**

The ratio of the anteroposterior to mediolateral diameters of the proximal femur was significantly greater in the TRO group (*p* < 0.01). The thicknesses of the muscles did not differ between the two groups, whereas the anterior capsule was significantly thicker in the TRO group (*p* < 0.05). Varus or valgus stem alignment (> 3°) was frequent in the TRO group (*p* < 0.01).

**Conclusions:**

The round shape of the proximal femur was deformed after TRO compared with primary THA for ONFH, which may have caused malposition of the stem. In addition, we should pay attention to anterior protrusion of the proximal femur and thickening of the anterior capsule.

## Background

One of the surgical treatments for osteonecrosis of the femoral head (ONFH), which frequently occurs in adolescence [1, 2], is transtrochanteric rotational osteotomy (TRO) [3–8]. In this procedure, the femoral head is rotated anteriorly or posteriorly to move the healthy bone to the weight-bearing area and the necrotic bone to the non- weight-bearing area [3–8]. Some studies have demonstrated good clinical outcomes of TRO [3–8]. However, if the femoral head collapses or secondary osteoarthritic changes appear after surgery, total hip arthroplasty (THA) is needed. In patients who undergo THA after failed TRO, morphological changes in the proximal femur can occur, which can lead to malalignment of the insertion of the femoral component. Muscle atrophy and soft tissue thickening of the hip joint can also occur, which can lead to reduced centripetal force and anterior impingement. These are risk factors for posterior dislocation and require attention. According to some reports, proper treatment for the anatomic changes in the proximal femur and removal of sclerotic lesions in the femoral canal are essential for the success of conversion THA [9–16]. However, few reports have clarified the degree of bony morphological deformity of the proximal femur, muscle atrophy, and soft tissues after TRO [17, 18]. The purposes of this study were (1) to measure the morphological deformity of the proximal femur, muscle atrophy, and thickness of the anterior soft tissue in patients who underwent THA after failed TRO and in those who underwent primary THA for ONFH and (2) to determine the effects of conversion THA on clinical outcomes.

## Methods

This retrospective cohort study was approved by our institutional review board (approval no. 1843) and included 17 patients who underwent THA after failed TRO at our hospital between 2000 and 2015 (TRO group: 18 hips). The mean age of the TRO group was 40.8 years (range, 18–58 years), and the mean time from TRO to THA was 52.2 months (range, 14–252 months). We also included a study group of 21 patients who underwent primary THA for ONFH during the same period (control group: 28 hips). The mean age of the control group was 42 years (range, 21–68 years), and the control group included the Japanese Investigation Committee classification stage 3A:1 hip, stage 3B: 13 hips, and stage 4: 14 hips. All patients were available for follow-up for at least 2 years after THA. Patient information was obtained from medical records, and the data were routinely collected. All procedures in both groups were performed using the posterior approach. In the TRO group, cementless femoral components were used in 16 hips and cemented components in 2 hips; in the control group, all femoral components were cementless (Table 1). In all cases, the acetabular component was cementless.

**Table 1:** Patient demographics

**Characteristic**	TRO group (18 hips)	Control group (28 hips)	*p* value
Number of patients	17	21	
Mean age, years (SD)	40.8 (11)	42 (14)	0.76
Gender (male/female)	16/1	16/5	0.20
Mean BMI, kg/m^2^ (SD)	23.5 (3.3)	22.1 (4.4)	0.24
Mean length of follow-up, months (SD)	78.9 (45)	106.3 (39)	< 0.05*
Etiology of ONFH			
Steroid/alcoholism/idiopathic	8/ 8/ 1	14/ 7/ 0	0.77
Approach: posterior (n)	18	28	
**Femoral component**			
Cementless (n)	16	28	0.15
Cement (n)	2	0	

*BMI* Body mass index, *SD* Standard deviation, *TRO* Transtrochanteric rotational osteotomy

^*^Statistically significant

In implants information, in the TRO group, four designs of cementless implants were used, depending on when the THA was performed: a Trident PSL cup with a Super Secur-Fit stem and Trident crossfire liner (Stryker; Kalamazoo, MI, USA) from 2000 to 2006, a Trilogy with a VerSys Mid-coat and Longevity (Zimmer Biomet; Warsaw, IN, USA) from 2007 to 2008, an ADEPT cup with a Super Secur-Fit (Stryker; Kalamazoo, MI, USA) from 2009 to 2010, and an AMS HA cup with a J-taper and AQUARA (Kyocera, Osaka, Japan) from 2012 to 2015. The two hips of the cement stem were used by Exeter and Omnifit stem (Stryker; Kalamazoo, MI, USA). The head size depended on both the periods of THA and cup size. A 26-mm zirconia ball was used in 5 hips. 5 hips were treated with a 28-mm zirconia or alumina ball, 5 hips were treated with a 32- mm zirconia or alumina ball, and 3 hips were treated with a 42–44 mm CoCr ball.

### Surgical procedure for THA after TRO

After the induction of general anesthesia, each patient was placed in the lateral decubitus position. Through the posterior approach, an incision was made in the fascia of the iliotibial band, and the adherent gluteus medius muscle was released. An incision was made posterior to the vastus lateralis, which was retracted anteriorly toward the femur, and the osteosynthesis plate and screws were removed. There was no piriformis muscle or short external rotator muscle during the posterior approach to the hip joint; scar tissue was present in their place. The posterior articular capsule was resected, and the femoral head was exposed. The hip was flexed and internally rotated to dislocate the femoral head, and osteotomy of the femoral neck was performed. The surface of the osteotomy level was round, and the lag screw corridor in the proximal femur showed hard osteosclerosis. The thickened anterior articular capsule was resected, the hip was reduced to a neutral position, and the femur was anteriorly retracted with acetabular retractors for acetabulum exposure. The acetabulum was reamed, and the cup was press-fitted with a cup inclination angle of 40° and a cup anteversion angle of 15°.

Next, the femoral canal was prepared. The hip was flexed and rotated internally to expose the proximal femur. There was no iliopsoas tendon on the lesser trochanter. The osteotomy surface of the femur protruded anteriorly. The rasp of the femoral component was inserted as the osteotomy surface of the posterior wall. The osteosclerotic bones around the lag screw corridor were identified and shaved with a high-speed stainless-steel burr, and a straight starting reamer was inserted. We then used an image intensifier to confirm the stem alignment. The rasp was placed with an antetorsion angle of 30° with reference to the lower leg axis. The anteriorly protruding bones were resected, the hip joint was reduced, and the range of motion was checked for impingement. Then,, the femoral component was placed. Full weight bearing was allowed from the day after surgery.

### Clinical and radiological evaluation

**Fig. 1:**
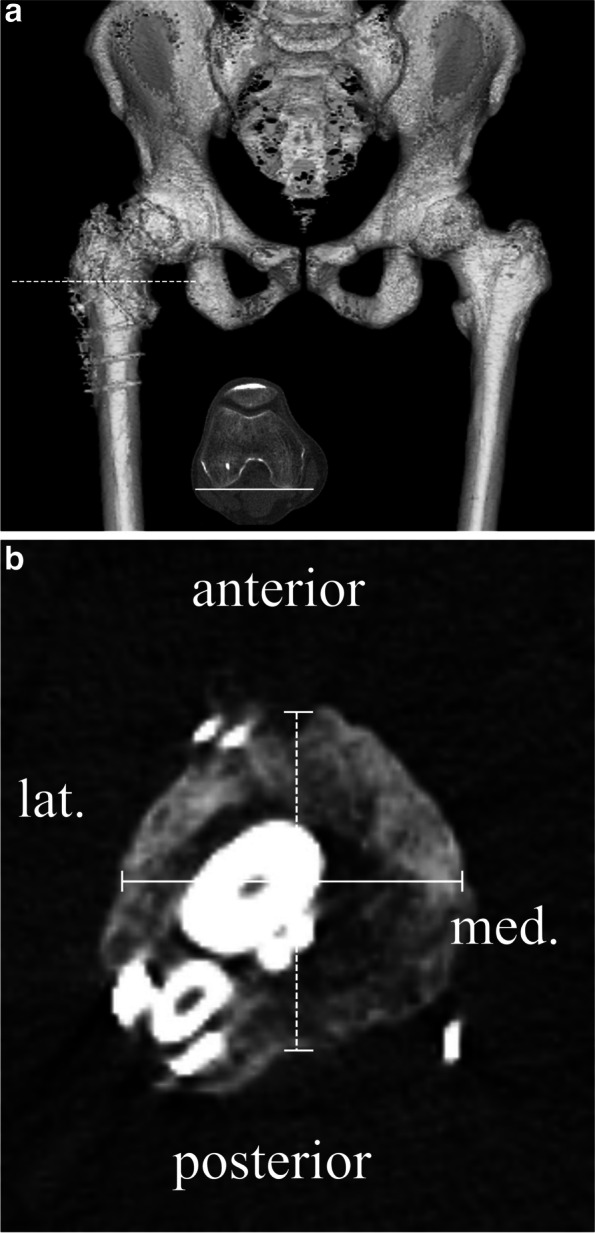
Evaluation of the deformed proximal femur. **a** The reference plane was defined as the plane passing through the proximal most posterior point of the femur (white line) and bilateral posterior femoral condyle. The location of the measurement was the axial image sliced 5 mm proximal from the lesser trochanter (white dotted line). **b** The ratio of the anteroposterior and mediolateral diameters of the femur was calculated by measuring the anteroposterior (white dotted line) and mediolateral diameters (white line) of the femur in the axial image sliced

**Fig. 2:**
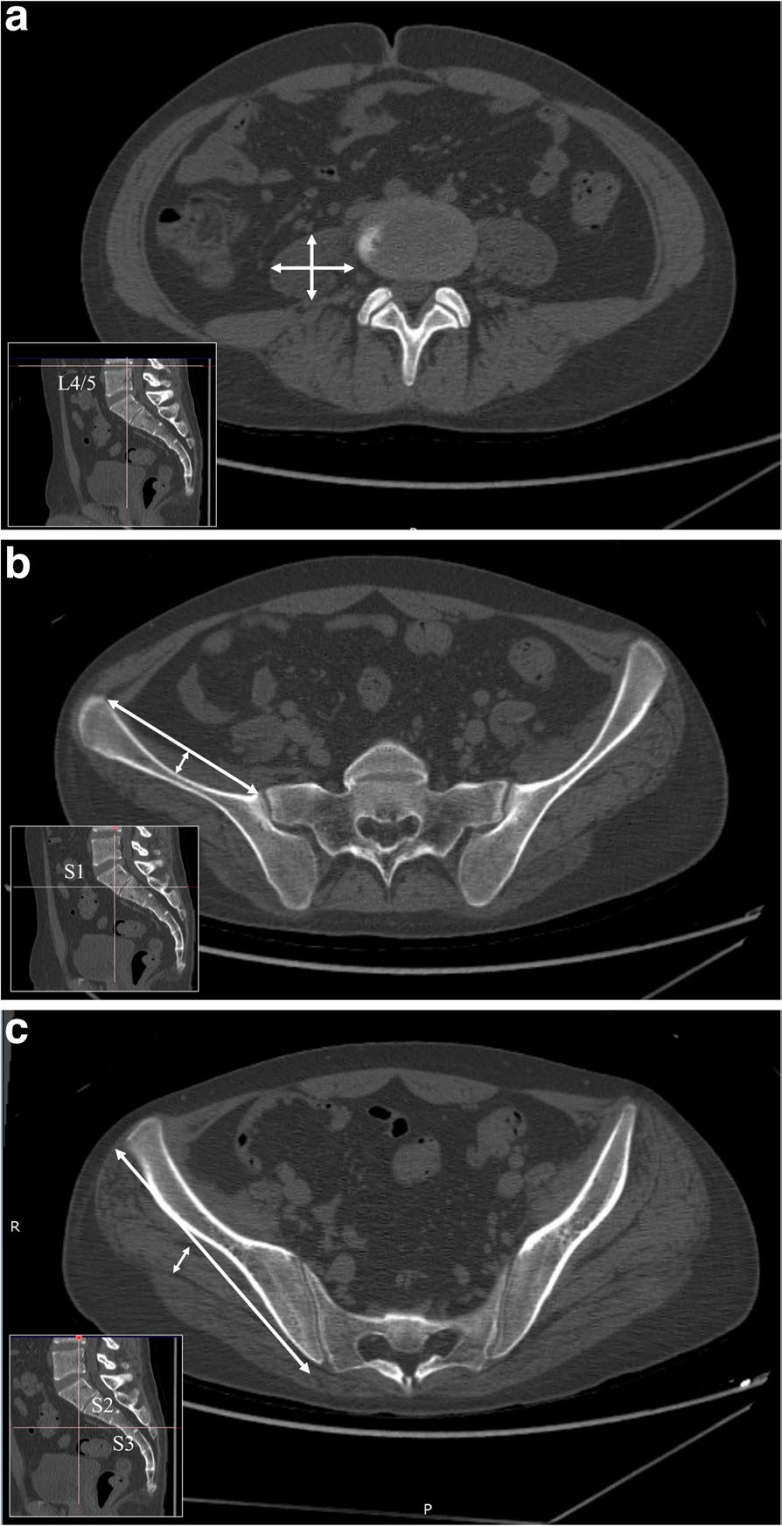
Evaluation of muscle atrophy. **a** For the psoas major muscle belly, we calculated the ratio of the anteroposterior and mediolateral diameters at the level of the L4/5 intervertebral disc. **b** For the iliac muscle belly, we calculated the ratio of the maximum thickness and width at the S1 level. **c** For the gluteus medius muscle belly, we calculated the ratio of the maximum thickness and width at the S2–S3 level

**Fig. 3:**
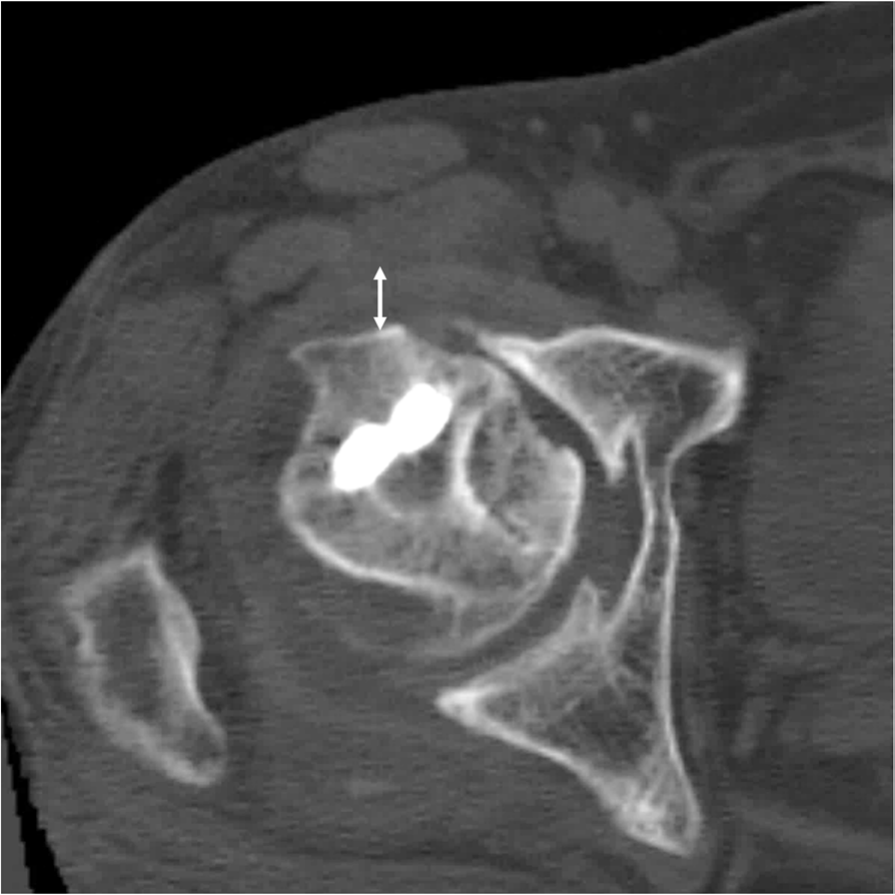
Evaluation of the thickness of anterior articular capsule. The capsule thickness was measured on the axial image at the level of the center of the femoral head

The clinical parameters evaluated included operative time, blood loss, and the presence of complications. We used the Japanese Orthopaedic Association (JOA) hip score to perform clinical assessments preoperatively and at the latest follow-up [19]. The JOA hip score is the total of scores on four subscales for pain (score range: 0–40), range of movement (0–20), gait (0–20), and activities of daily living (0–20). The maximum total score of 100 points represents the absence of symptoms. The JOA hip score has been shown to be correlated with the Harris hip score [19, 20]. JOA scores were collected for all patients in the hospital, and JOA scores for THA after failed TRO and primary THA for ONFH were compared. We used three-dimensional analysis software (ZedHip^Ⓡ^; LEXI Co., Ltd., Tokyo, Japan) for radiological evaluation to assess the deformity of the proximal femur. Using preoperative computed tomographic (CT) images of the THA (GE Light Speed QX/i with 2.5-mm slice thickness or GE medical system Light Speed VCT with 1.25-mm slice sickness or Canon medical system, Aquilion ONE, 1-mm slice thickness), we created a reference plane of the femur called “the table-top plane” that passing through the most proximal posterior point of the femur and the bilateral posterior femoral condyle [21, 22] (Fig. 1a). To evaluate the three-dimensional bony morphological features of the femur, we calculated the ratio of the anteroposterior and mediolateral diameters of the femur by measuring the anteroposterior and mediolateral diameters in the axial CT slice 5 mm proximal from the lesser trochanter (Fig. 1b). To evaluate muscle atrophy, the thicknesses of the psoas major, iliac, and gluteus medius muscles on the operated side were measured on the preoperative CT axial image. We have modified the method for measuring muscle volume [23] and calculated the ratio of the anteroposterior and mediolateral diameters of the psoas major muscle belly at the level of the L4/5 intervertebral disc, the ratio of the maximum thickness to width of the iliac muscle belly at the S1 level, and the ratio of the maximum thickness to width of the gluteus medius muscle belly at the S2–S3 level (Fig. 2a–c). The thickness of the anterior articular capsule was also measured on the axial image at the level of the center of the femoral head (Fig. 3). To evaluate the alignment of the components, we assessed the cup inclination and anteversion angle using radiographic definition. We also assessed the stem anteversion angle, and presence of stem varus or valgus alignment > 3°, stem extended alignment > 3° or flexed alignment > 3° with reference to the axis of the proximal femur on the postoperative CT images.

### Statistical analysis

Statistical analyses were performed using JMP 15 (SAS Institute, Cary, NC, USA). We used the Mann–Whitney *U* test to compare continuous variables (surgical time and thicknesses of the iliac muscle, gluteus medius muscle, and anterior articular capsule) and Fisher’s exact test to compare categorical variables (including rate of postoperative dislocation and femoral component alignment) between the two groups.

## Results

Out of 18 hips, 16 hips had plate and screws removed simultaneously. The remaining 2 hips had the screws removed prior to THA. There were no significant differences in age, gender, and body mass index of the two groups (Table 1). We found no difference in JOA scores and hip ranges of motion between the two groups at the latest observation. In the TRO group, operative time was significantly longer (means 203 vs. 123 min, respectively) and blood loss was significantly greater (means 717 vs. 365 mL, respectively; *p* < 0.01) compared with the control group. In terms of complications, posterior dislocation was observed in 4 hips (22%) in the TRO group and 1 hip (4%) in the control group; the incidence of dislocation was not significantly different between both groups (*p* = 0.07, according to Fisher’s exact test; Table 2). With regard to deformity of the proximal femur, the anteroposterior diameter of the proximal femur was significantly larger in the TRO group (*p* < 0.01, according to an unpaired *t* test), but the mediolateral diameters of the two groups did not significant difference. The mean ratio of anteroposterior to mediolateral diameters was significantly larger in the TRO group (1.01 [standard deviation (SD) 0.1]) than in the control group (0.79 [SD 0.1]); the proximal femur protruded anteriorly in the TRO group, and its cross-sectional appearance had a 1:1 aspect ratio (Table 3).

**Table 2:** Treatment outcomes

**Outcome**	TRO group	Control group	*p* value
Mean surgical time, min (SD)	203 (82)	123 (40)	< 0.01*
Mean blood loss, mL (SD)	717 (464)	365 (226)	< 0.01*
**Complication**			
Posterior dislocation (n)	4	1	0.07
Infection (n)	0	0	
Fracture (n)	0	0	
**Mean JOA score (SD)**	91.3 (8.5)	86.2 (21.2)	0.28
**Mean ROM (SD)**			
Flexion	104° (14°)	111° (11°)	0.09
Abduction	35° (6°)	35° (8°)	0.97
External rotation	39° (13°)	42° (13°)	0.52
Internal rotation	19° (9°)	22° (10°)	0.37

*JOA* Japanese Orthopedic Association, *ROM* Range of motion, *SD* Standard deviation, T*RO* Transtrochanteric rotational osteotomy

^*^Statistically significant

**Table 3:** Shape of the proximal femur

**Measurement**	TRO group	Control group	*p* value
Mean AP diameter, mm (SD)	50.0 (7.0)	37.2 (3.8)	< 0.001*
Mean ML diameter, mm (SD)	49.9 (5.5)	47.2 (3.8)	0.12
Mean AP/ML ratio (SD)	1.01 (0.1)	0.79 (0.1)	< 0.001*

*AP* Anteroposterior, *ML* Mediolateral, *SD* Standard deviation, *TRO* Transtrochanteric rotational osteotomy

^*^Statistically significant

In evaluating muscle atrophy on the operated side, we found no differences between the two groups in the anteroposterior and mediolateral diameters of the psoas major or in the ratio of the thicknesses and widths of the iliac and gluteus medius muscles (Table 4). With regard to soft tissue thickening, the anterior articular capsule was significantly thicker in the TRO group (mean 10.3 mm) than in the control group (mean 8.1 mm; *p* < 0.05, according to an unpaired *t* test; Table 5). In evaluating component alignment on postoperative CT images, we found that the mean cup inclination angle was significantly larger in the control group (42.1°) than in the TRO group (36.8°; *p* < 0.01, according to an unpaired *t* test; Table 6), but the cup anteversion angles and stem antetorsion angles of the two groups did not differ. In the coronal alignment of the stem, varus or valgus stem alignment (> 3°) was observed in 7 hips (39%; 3 varus and 4 valgus) in the TRO group but in only 1 hip (4%; one varus) in the control group, and stem malalignment was significantly worse in the TRO group (*p* < 0.01, according to Fisher’s exact test; Table 6). In contrast, the sagittal alignment of the stem did not differ in the two groups.

**Table 4:** Muscle atrophy on the operated side

**Muscle**	TRO group	Control group	*p* value
**Psoas major**			
Mean AP diameter, mm (SD)	28.0 (5.8)	31.2 (3.1)	0.09
Mean ML diameter, mm (SD)	28.7 (5.4)	31.7 (6.6)	0.20
Mean ratio of the AP and ML diameters (SD)	0.99 (0.23)	1.04 (0.31)	0.69
**Iliac**			
Mean thickness, mm (SD)	8.9 (2.9)	9.0 (3.3)	0.87
Mean width, mm (SD)	82.1 (4.3)	83.9 (6.6)	0.38
Mean thickness/width ratio (SD)	0.11 (0.03)	0.11 (0.04)	0.88
**Gluteus medius**			
Mean thickness, mm (SD)	24.0 (7.0)	27.3 (5.1)	0.14
Mean width, mm (SD)	123.4 (9.9)	123.2 (10.6)	0.96
Mean thickness/width ratio (SD)	0.20 (0.06)	0.22 (0.04)	0.25

*AP* Anteroposterior, *ML* Mediolateral, *SD* Standard deviation, *TRO* Transtrochanteric rotational osteotomy

**Table 5:** Thickness of the anterior articular capsule

**Characteristic**	TRO group	Control group	*p* value
Mean thickness of the anterior articular capsule, mm (SD)	10.3 (3.0)	8.1 (2.6)	< 0.05*

*SD* Standard deviation, *TRO* Transtrochanteric rotational osteotomy

^*^Statistically significant

**Table 6:** Alignment of the cup and stem of the femoral head

**Characteristic**	TRO group	Control group	*p* value
Mean cup inclination (SD)	36.8° (5.6°)	42.1° (5.7°)	< 0.01*
Mean cup anteversion (SD)	14.9° (5.0°)	12.6° (5.9°)	0.23
Mean stem anteversion (SD)	18.3° (11.1°)	20.5° (18.9°)	0.71
Varus alignment > 3°, n	3	1	< 0.01*
Valgus alignment > 3°, n	4	0	
Extended alignment > 3°, n	2	2	0.23
Flexed alignment > 3°, n	3	1	

*SD* Standard deviation, *TRO* Transtrochanteric rotational osteotomy

^*^Statistically significant

### Case presentation

**Fig. 4:**
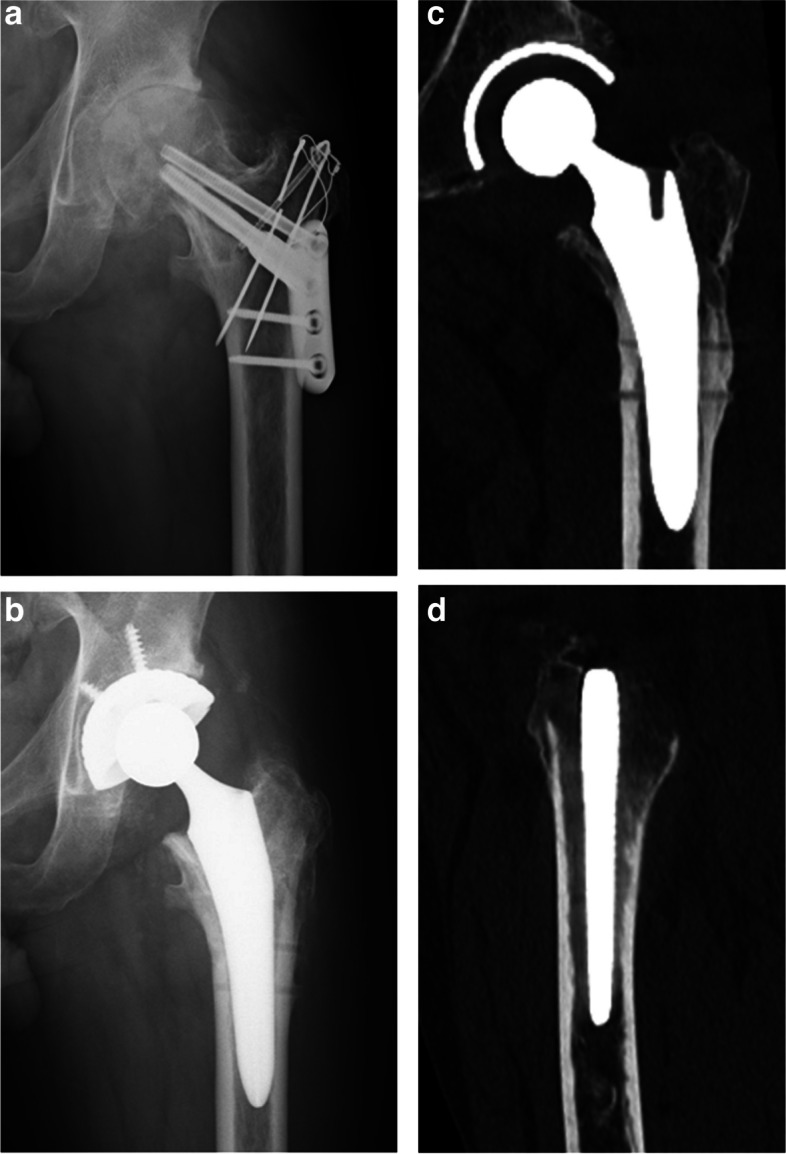
Fifty-four-year-old man with osteonecrosis of left femoral head related alcohol. **a** Radiological appearance of the left hip 11 years after transtrochanteric rotational osteotomy (TRO). **b** Radiological appearance after total hip arthroplasty (THA). **c** Postoperative CT coronal image showed 4° of varus alignment of the stem and osteosclerosis around the lag screw corridor in the medullary cavity. **d** Postoperative CT sagittal image showed 1° of extended alignment of the stem

The patient was a 54-year-old man with osteonecrosis of left femoral head related alcohol. TRO surgery was performed at age 43, however osteoarthritis subsequently progressed (Fig. 4a). THA was performed using a cementless implants (an AMS HA cup with a J-taper stem and AQUARA liner) (Fig. 4b). Postoperative CT image showed 4° of varus alignment of the stem and osteosclerosis around the lag screw corridor in the medullary cavity (Fig. 4c). Postoperative CT sagittal image showed 1° of extended alignment of the stem (Fig. 4d).

## Discussion

During the 2-year follow-up of this study, the JOA scores of patients in the TRO group were not significantly worse than those of the control group, but the TRO group had more complications. THA after failed TRO has been reported in the past to be associated with longer operative time and more blood loss than primary THA^9, 10, 12,15)^, and stem malalignment has also been reported ^9–16)^.

We found that the TRO group caused more stem malalignment and tended to have a higher rate of posterior dislocation than the control group. These outcomes may have resulted from morphological changes in the proximal femur. With regard to such deformities in patients undergoing THA after failed TRO, some authors have reported that orienting the stem insertion is difficult, and anterior protrusion of the rotational bone fragments can result in anterior impingement [9, 11–13, 17, 18]. In our TRO group, we also found that the proximal femur protruded anteriorly and appeared round in cross-sectional images, and the anterior articular capsule was significantly thickened. These deformities were thought to have resulted in stem insertion error, which led to stem malalignment and anterior impingement; these may result in posterior dislocation. At our institution, we evaluated the anteriorly protruding bone and thickened anterior articular capsule preoperatively using three-dimensional templating software (ZedHip®) and resected them intraoperatively. As a result, anterior impingement did not occur, and there may have been no significant difference in dislocation. In addition, in patients who undergo THA after failed TRO, intramedullary abnormalities such as hard osteosclerosis around the lag screw corridor are often observed in the medullary cavity. Some authors have also reported the need to use a steel burr to penetrate such areas in preparation for femoral reaming [9, 11–13].

We found no significant difference in muscle atrophy of the psoas major, iliac, and gluteus medius muscles between the two groups. The patients in the control group were unable to walk with sufficient weight bearing before surgery because of pain, and they probably had the same type of muscle atrophy as in the TRO group; thus, such patients should be compared with people with intact hips.

Among the femoral components used in the TRO group, 16 stems were cementless and 2 were cemented. At our institution, we are currently using (ZedHip®) to implant a stem that fits in the medullary cavity of the femur. The structure of the proximal femur is complex in patients who undergo THA after failed TRO; thus, if the stem does not fit precisely in the medullary cavity, the use of a cemented stem or a distally fixed stem should be considered.

We suggest several tips to ensure the success of THA after failed TRO. First, surgeons must be aware that the structure of the proximal femur is abnormal and that the anterior articular capsule is thickened. The stem insertion point should be noted on the anteriorly protruding bone at the proximal femur as the osteotomy surface of the posterior wall. For optimal stem alignment, the intramedullary area of hard osteosclerosis should be shaved with a steel burr to prevent bone perforation, and stem alignment should be confirmed intraoperatively using an image intensifier.

Fortunately, no intraoperative fractures or stem perforations occurred in this study. However, deformities in the proximal femur and osteosclerosis around the lag screw corridor, as well as the holes from the removal screw insertion after TRO, may increase the risk of not only stem malposition but also periprosthetic femoral fractures [24] and stem perforation [25] at the removal screw holes. Hence, meticulous preoperative planning and careful intraoperative techniques are considered essential.

There is still considerable methodological consideration regarding the heights from the lesser trochanter for deformity analysis. Our results showed and discussed about the accuracy of stem placement and surgical techniques. Therefore, it seems necessary for future research to conduct deformation analysis that replicates the actual surgical field, including the resection plane of the femoral neck calcar at the total hip arthroplasty.

This study had several limitations. First, it was retrospective. Second, the number of cases was relatively small (18 hips). Third, the control group consisted of patients who had undergone primary THA for ONFH; patients who underwent THA after TRO may need to be compared with those whose hips are intact. If THA is to be performed after failed TRO, we should recognize that the proximal femur is deformed and recommend careful preoperative planning and the use of a steel burr and an image intensifier to avoid stem malalignment.

## Conclusions

THA after failed TRO was more complicated than primary THA. Clinical scores were not significantly different between patients who had previously undergone TRO and those who had undergone primary THA; however, stem malalignment was observed in the TRO group. Deformities of the proximal femur after TRO include roundness and hard sclerotic bones around the lag screw corridor, which hinder the accurate determination of the stem insertion point and facilitate canal preparation and cause stem malalignment.

## Data Availability

All data used and analyzed during the current study are available from the corresponding author on reasonable request.

## Abbreviations

CT: Computed tomographic
JOA: Japanese Orthopedic Association
ONFH: Osteonecrosis of the femoral head
SD: Standard deviation
THA: Total hip arthroplasty
TRO: Transtrochanteric rotational osteotomy
